# Assessment of Rational Design of Self-Compacting Concrete Incorporating Fly Ash and Limestone Powder in Terms of Long-Term Durability

**DOI:** 10.3390/ma13122863

**Published:** 2020-06-26

**Authors:** Pavel Reiterman, Roman Jaskulski, Wojciech Kubissa, Ondřej Holčapek, Martin Keppert

**Affiliations:** 1 University Centre for Energy Efficient Buildings, Czech Technical University in Prague, Třinecká 1024, 273 43 Buštěhrad, Czech Republic; 2 Faculty of Civil Engineering, Czech Technical University in Prague, Thákurova 7, 166 29 Prague, Czech Republic; ondrej.holcapek@fsv.cvut.cz (O.H.); martin.keppert@fsv.cvut.cz (M.K.); 3 Faculty of Civil Engineering, Mechanics and Petrochemistry, Warsaw University of Technology, Lukasiewicza St. 17, 09-400 Plock, Poland; Roman.Jaskulski@pw.edu.pl (R.J.); Wojciech.Kubissa@pw.edu.pl (W.K.)

**Keywords:** self-compacting concrete, fly ash addition, limestone powder, durability, carbonation

## Abstract

Self-compaction concrete (SCC) is ranked among the main technological innovations of the last decades. Hence, it introduces a suitable possibility for further utilization of supplementary cementitious materials (SCM) in terms of sustainable development. The aim of the work is the assessment of a new approach to binder design, which takes into consideration the activity of the used mineral additive. The proposed approach, which allows a systematic design of a binding system with varied properties of the used mineral additive, was studied on ternary blends consisting of Portland cement (PC), limestone powder and fly ash (FA). The verification was conducted on SCC mixtures in terms of their workability, mechanical properties and the most attention was paid to long-term durability. The long-term durability was assessed on the basis of shrinkage measurement, freeze-thaw resistance and permeability tests including initial surface absorption, chloride migration, water penetration and an accelerated carbonation test, which was compared with the evolution of carbonation front in normal conditions. The durability of studied mixtures was evaluated by using durability loss index, which allow general assessment on the basis of multiple parameters. The carbonation resistance had a dominant importance on the final durability performance of studied mixtures. The experimental program revealed that the proposed design method is reliable only in terms of properties in fresh state and mechanical performance, which were similar with control mixture. Despite suitable results of freeze-thaw resistance and shrinkage, an increasing amount of fly ash in terms of the new design concept led to a fundamental increase of permeability and thus to decay of long-term durability. Acceptable properties were achieved for the lowest dosage of fly ash.

## 1. Introduction

Portland cement-based concrete is the most widely used structural material in the world, with an annual production around ten billion metric tons [1]. A crucial factor in terms of sustainability is the production of Portland cement; it is responsible for 5–7% of global CO_2_ emissions [2]. According to Habert [3], the annual production of cement was 2.8 billion tons in 2014. Schneider et al. [4] predicted that production would exceed 4 billion tons by 2050; however, according to USGS, global cement production already amounted to 4.1 billion tons in 2018. The production of one metric ton of cement releases an estimated 0.73–0.99 t of CO_2_ depending on the ratio of the clinker and used fuel [5]. Hence, the reduction of the negative impacts of cement production has become a global effort [6,7,8,9]. The replacement of cement by other cementing materials is the main approach to meet the required CO_2_ reduction [10,11]. In addition, the application of supplementary cementitious materials (SCM) could be beneficial in terms of durability and other engineering requirements [12,13,14]. The more environmentally-friendly nature of such concrete results from the reduction of CO_2_ emission associated with the production of cement clinker [15] and other factors negatively influencing the sustainability of the building industry.

Fly ash (FA), which has proper standard properties in accordance with the BS EN 450-1 [16] standard, can be used in cement and concrete manufacture. However, the properties of this material strictly depend on the kind of combusted solid fuel and technology of combustion used, and it varies during the year [17,18,19]. The result is that some types of FA do not meet adequate properties for typical utilization in binding mixture [20,21]. For example, fluidized combustion installations generate by-products which have significantly different properties compared to those arising in pulverized combustion. Thus, FA coming from different sources may significantly differ in their chemical and physical properties, which have different impacts on hydration processes in cement–fly ash binder [18,22]. The pozzolanic activity of FA can be enhanced using some chemical admixtures (NaOH, Na_2_SO4 and Na_2_CO_3_) [23]. The positive influence of FA on durability was declared by a number of studies [24,25] and practical experiences [26], because of the slower reaction kinetics of FA hydration contributing to the gradual densifying. However, global interest is focused on its influence in presence with other SCM [27,28], especially with limestone filler [29,30].

Limestone powder (LS), according to CSN EN 206-1 [31], is an inert mineral additive which does not attend the process of PC hydration. However, Matschei et al. [32] reported a double role of limestone powder—besides its physical effect leading to the densifying of internal structure, its presence causes formation of new hydration products. It was reported that limestone powder reacts primarily with calcium aluminate hydrates [33] to form monocarbonate aluminate, which is an analog of monosulfoaluminate. The formation of this additional stable phase AFm tightens the concrete structure, reducing its porosity [34,35,36]. On the other hand, derived optimal proportions of ternary systems always reflect actual composition and given properties of used components, which could be highly variable.

Current concrete production prefers automatic technologies, which limit the amount of human labor on the construction site. Hence, self-compacting concrete (SCC) mixtures are widely used for a number of applications. SCC was developed primarily for exposed, highly reinforced concrete structures. The required workability of SCC is determined by the higher amount of fine particles to achieve suitable mobility of fresh mixture [37,38], especially cement and mineral additives. The denser structure of such concrete prevents the ingress of aggressive media, especially when low water to binder ratio is used [39,40]. On the other hand, the high content of fine particles could cause a significant increase of creep and shrinkage [41], which lead to the initiation of cracks. Despite the density of the concrete with a high cement replacement by FA, the depth of carbonation increases at given binder content [42]. However, this study reported that, in the case of higher amount of FA, the carbonation depth corresponding to structural concrete could be achieved by significant reduction of w/b and prolonged curing. Without these measures, high-volume fly ash concrete (HVFAC) is inappropriate in terms of durability. 

The tightness of concrete, and therefore its resistance to carbonation, can also be related to its resistance to shrinkage cracking. Altoubat et al. [25] analyzed the resistance to shrinkage cracking of SCC containing 20%, 30% and 50% of FA. The concrete was subjected to three different curing regimes: air drying, three-day moist curing and seven-day moist curing. The results indicate that the use of up to 35% FA does not lead to a reduction in the resistance of the concrete to shrinkage cracking, provided that it was moist cured by at least three days.

Siddique [24] examined SCC with a binary binding system incorporating class F fly ash content from 15 to 35 wt.%. One of the parameters studied was the depth of carbonation. The results follow a general trend, from which it can be concluded that, with the increase in the amount of FA used, the depth of carbonation increases after 365 days of testing. However, the largest depth of carbonation after 365 days was 1.85 mm, which indicates a low rate of this process. The tendency of HVFAC to reduce resistance to carbonation is caused by the significant reduction of pH and slower kinetics of hydration, which is in fact competitive to the carbonation.

The drawbacks of fly ash such as delayed setting could be effectively compensated by the addition of limestone powder [43], which contributes to the particle packing and hydrates precipitation [32]. When it is used in an amount higher than 5%, it usually leads to a significant increase in the viscosity of the mixture and deterioration of its workability [30,44]. In addition, Duran-Herrera et al. [45] reported reduction of shrinkage using replacement from 5 to 15 wt.% of fly ash by limestone powder.

Celik et al. [30] showed that the replacement of a large amount of PC (up to 55% by weight) by fly ash or the fly ash with the simultaneous addition of limestone powder allows obtaining workable, high strength SCC without the use of viscosity correction additives. It was shown that after 365 days the SCC with FA and 25% or 15% limestone addition had greater strength than SCC with only limestone in the same amount. However, concrete with studied additive without limestone proved to be more durable than the FA and limestone SCC. However, limestone used in this study was coarser in comparison with studied pozzolanic additive, hence exhibited negative impact.

Boel et al. [46] analyzed the effect of the composition of SCC on their transport properties, including gas permeability. When limestone filler was replaced by fly ash in one concrete mixture, the results show that FA contributes more to the sealing of the structure of concrete than limestone filler in the case of concrete with the same material parameters (amount and type of cement, w/c ratio, binder to filler ratio); however, appropriate composition modifications of SCC with limestone filler, e.g., reducing the w/c ratio from 0.46 to 0.40, can result in concrete with a clearly lower apparent gas permeability value than FA concrete with the unchanged initial composition.

Gesoglu et al. [47] performed a comprehensive study of the fresh and hardened characteristics of binary and ternary SCC mixtures containing FA, marble and limestone filler. Of the twelve tested mixtures, three were the ternary ones based on cement, FA and limestone filler. Compared to the control mixture containing PC alone, these mixtures were characterized by a lower slump flow time *t*_500 mm_, which additionally showed a decreasing tendency with the increasing share of limestone filler replacing cement. The flow of fresh mixtures also increased, but only the addition of 15% limestone filler allowed a higher flow than for the reference mix. Both results were obtained by using about 50% less superplasticizer in relation to the reference mixture.

Application of various SCM presents an efficient approach to limit the negative impacts of cement production; however, the nature of the material has to be taken into consideration due to the high variability of a single SCM. Ternary binding systems are currently studied intensively, because they allow compensating for possible negative impact of selected mineral additives. However, the optimal proportions of binding system significantly vary with the properties of the used binder components, water to binder ratio and other aspects of concrete mixture composition; thus, optimal binder proportions could differ one from another. The studied design procedure allows a simple tool for a systematic formulation of binding system. SCC mixtures based on the ternary binder system were selected for the verification of a new approach for the design of concrete composition which takes into the consideration the amount of amorphous phases in used SCM. This work presents application of this concept in the case of SCC mixtures incorporating fly ash and limestone filler, the dosage of which was reduced with an increasing amount of the used fly ash. The applicability of the proposed mixture design was verified in terms of selected durability properties. This new concept could also be applied in the case of other alternative active mineral additives, whose importance is constantly growing due to the requirements for the sustainability of building industry. 

## 2. Materials and Methods 

The performed experimental program was focused on the rational design of self-compacting concrete incorporating limestone filler and fly ash as partial cement replacement. The main aim of the program was to take into consideration the reactivity of used FA, as well as the part of used additive resulting in the pozzolanic reaction, and the verification of this approach in terms of complex system of methods relating to the durability. The chemical composition determined by XRF (Thermo ARL 9400 XP, Basel, Switzerland) of used binder components is shown in Table 1.

**Figure 1:**
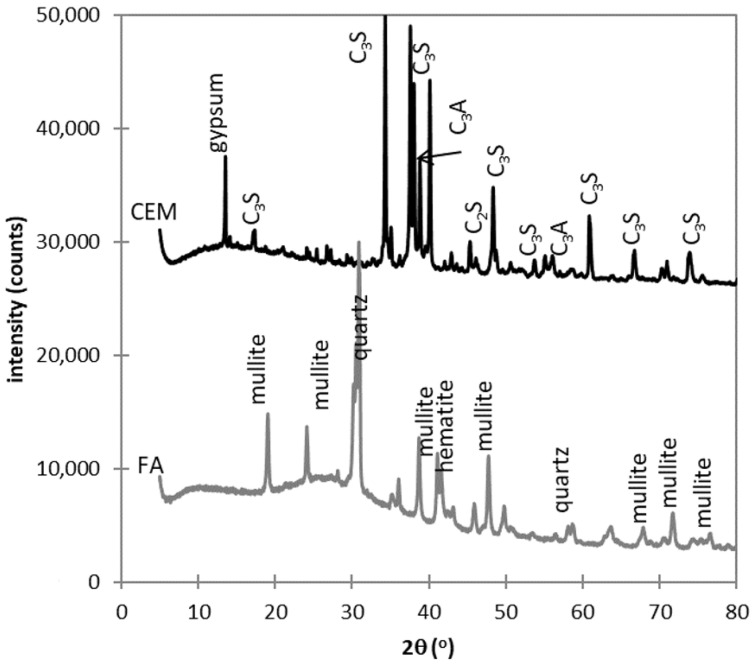
X-ray diffractograms of used cement and fly ash.

The XRD analysis of raw materials was performed using a PAnalytical Aeris diffractometer (Malvern Panalytical, Malvern, UK) equipped with a Co_Kα_ tube operating at 40 kV and 7.5 mA. The detector used was a PIXcel^1D^-Medipix3 detector (Malvern Panalytical) with an active length of 5.542°. The scan ranged from 5° to 85°, step size was 0.0027°, and counting time was 2.0325 s. Data were evaluated by Profex software (version 3.12.1) [48]. The amount of amorphous portion was determined using an added internal standard (20% of ZnO). The diffractograms obtained are illustrated in Figure 1. 

**Figure 2:**
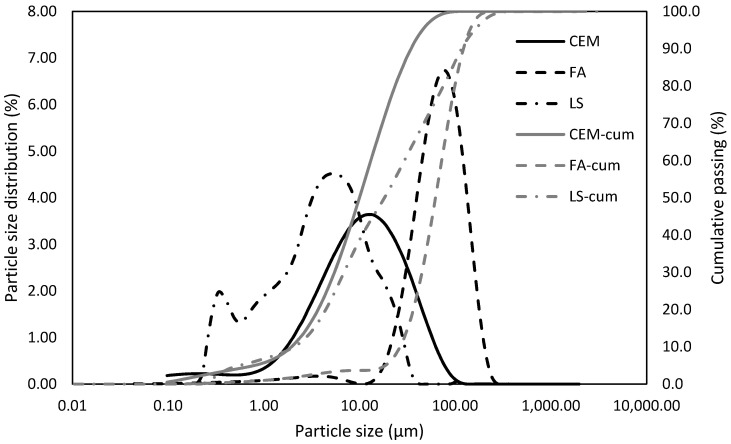
Particle size distribution of used binder components.

Mixture design. Rational design of binder system is based on consideration of the phase composition of the used active mineral additive. The used fly ash contains 60% of amorphous phase (Table 2), which may participate in the pozzolanic reaction, and the residue serves as filler. This design philosophy was applied by Keppert et al. [49], who studied application of ceramic powder exhibiting pozzolanic activity, and the inert part of used additive was compensated by the reduction of the dosage of sand. However, this design concept has not been applied in the case of ternary binding system yet. The significant advantage of this concept is easy application on the other types of mineral additives with potential to pozzolanic reaction. Thus, cement was replaced by only amorphous share of the fly ash; the filler share of used FA was compensated by the reduction of limestone filler. The cement replacement by amorphous phases of FA was gradually increased up to 30%. Thus, the higher was the dosage of fly ash, the lower was the dosage of limestone filler. The used limestone filler exhibited D_50_ 17 µm. The content of CaCO_3_ is declared at 99.2%, with the rest formed by MgCO_3_. D_50_ of used Portland cement CEM I 52,5 R was 10 µm. Used fly ash can be characterized by D_50_ 62 µm and by pozzolanic activity through Chapelle test 627 mg Ca(OH)_2_/g. The detailed composition of studied SCC mixtures is shown in Table 3. The labeling of SCC mixtures followed the amount of replaced OPC; however, since only 60% of FA was supposed to be reactive, nearly 40% of FA amount replaced OPC. The proportions of single ternary system from the conventional point of view are introduced in Table 3. The particle size distribution was determined by the laser diffraction method (Figure 2).

The contents of natural siliceous sand of fraction 0–4 mm and crushed aggregate of fractions 4–8 and 8–16 mm were constant for all studied mixtures. The designed binder system was modified by the coupling of chemical admixtures, which ensured suitable consistency of fresh mixture. The carboxylic superplasticizer was used to achieve suitable workability without need for additional increase of mixing water. Prolonged workability is a frequent requirement for SCC; hence, a setting retarding agent was applied to capture the realistic nature of current SCC mixtures. The dosages of used chemical admixtures reflected the recommendations of their producer. Their doses were similar for all studied mixtures, as was the amount of mixing water. Single mixtures were prepared using a similar procedure: Firstly, all dry components were homogenized with half of the mixing water for 1 min in a horizontal laboratory mixer. After that, additives and the rest of the mixing water were added for 1 min. The complete batch was mixed for another 2 min. Samples were primarily cured in a water basin; however, their curing was interrupted in some cases according to the requirements of the testing procedures described below.

Three procedures, conventionally applied to SCC, were conducted for the analysis of the properties of fresh mixtures. The slump-flow, J-ring and L-box tests were performed in accordance with CSN EN 12350-8 [50], CSN EN 12350-12 [51] and CSN EN 12350-10 [52] respectively. The content of air in fresh concrete was monitored using the pressure method CSN EN 12350-7 [53].

Properties determined over time on studied concrete mixtures were tested by using three specimens of given dimensions. Detailed description of procedures applied on hardened concrete is provided in the following.

Mechanical properties were investigated in selected time interval in terms of compressive strength using 150 mm cubes and flexural strength (prisms 100 mm × 100 mm × 400 mm). The flexural test was organized as four-point bending with a support span of 300 mm and loads were located in the thirds of the span. Mechanical testing was conducted in accordance with CSN EN 12390-3 [54] and CSN EN 12390-5 [55].

An initial surface absorption test (ISAT) was conducted on cubic specimens in a selected time interval. This procedure serves well as an indicator of the durability of the concrete due to its easy realization and close relation to concrete permeability [56,57]. The testing apparatus is equipped with a glass capillary and scale, which allows the recording of the water flow (mL·m^−2^·s^−1^) in time. The value of water flow after 10 min is usually used as a basic surface quality indicator (Table 4) [56,57]. Due to the curing of samples in a water basin, the ISAT test was performed using cubes dried at 60 °C for 72 h, after which the cubes were left in the laboratory conditions for an additional 24 h.

A water penetration test was carried out in selected time intervals using cubic specimens. The sample is exposed on a loading area to the ingress of water pressure of 0.5 MPa for 72 h, and then the sample is broken by a split test and the maximal depth of penetrated water is measured CSN EN 1290-8 [58].

A chloride migration test was carried out in accordance to NT Built 492 [59] by utilizing three cylindrical specimens with a diameter of 100 mm and a height of 50 mm. The chloride migration coefficient in non-steady state (Dssm) is calculated according to the standard manual on the basis of the obtained depth of chloride penetration determined by 0.1 M solution of silver nitrate, test duration, applied voltage and temperature. The applied voltage and test duration depend on the initial response of each sample.

A frost resistance test was performed using prismatic specimens of 100 mm × 100 mm × 400 mm after 90 days of curing according to CSN 731322 [60]. The index of frost resistance introduces the decay of flexural strength after the prescribed number of freeze-thaw cycles. During the freezing period lasting 4 h, the saturated samples are cooled down to −18 °C; subsequently, the chamber is automatically flooded with water and the samples are kept for 2 h at 20 °C in a water basin. A freeze-thaw cycle finishes by the draining of the chamber and following the freezing period. Besides loss of mechanical performance, the potential loss of mass is also monitored, which is limited up to 5%. Therefore, samples are checked after every 25th cycle. The flexural test was done according to the above described procedure.

Shrinkage of the studied mixtures was studied by utilizing three cylindrical specimens with a diameter of 100 mm and a height of 200 mm. The testing was conducted in selected time intervals and for two ways of curing—in a water basin and aerial conditions. Each cylinder was equipped with a pair of metallic targets on its bases after demolding, which served for the fixation of the sample in the measuring stand with a conventional dial test indicator.

Accelerated carbonation test (ACT) follows instruction of CSN 13295 [61], which prescribes 1% of CO_2_ exposure for 56 days and approximately 70% of RH. Hence, sets of 100 mm × 100 mm × 400 mm prisms of each mixture were extracted from water basin after 90 days and subsequently kept in normal laboratory conditions for four weeks. After that, three samples of each mixture were inserted into the testing chamber, and the remaining ones were cured in normal aerial conditions and served as control set. The carbonation front was investigated in terms of a conventional colorimetric phenolphthalein test. This methodology was successfully applied in the previous research [62]. The results of carbonation are expressed by carbonation coefficient *K_field_* in accordance with Ficks first law (Equation (1)):

(1) $$d\left(t\right)=d_{0}+K_{field}\times \sqrt{t}$$

where *d(t)* is the depth of the carbonation front (mm) at time *t* of exposure (years)*, d_0_* is the initial value of the carbonation front (0 mm) and *K_field_* is the carbonation coefficient (mm/year^0.5^).

The service life prediction of reinforced concrete structures could be done with respect to necessary depassivation of embedded steel rebars. The characteristic depth of carbonation front *x_c,c_* could be derived from design penetration model in EHE-08 [63], as follows (Equation (2)):

(2) $$x_{c,c}\left(t_{SL}\right)=K_{field}\sqrt{\gamma_{f}\times t_{SL}}$$

where *γ_f_* is safety factor (*γ_f_* = 1.10 in EHE-08) and *t_SL_* is the design service life depending on the character and sense of the structure. Concrete structures belonging to key infrastructure such as bridges, highways and other civil structures have a prescribed design service life of 100 years. The present procedure for the assessment was used by Sáez del Bosque et al. [64].

The durability loss index (DLI) was calculated in order to evaluate the durability on the basis of all conducted tests (Equation (3)),

(3) $$\mathrm{DLI}=100\times \overset{n}{\underset{i=1}{\sum }}\frac{\alpha_{i}\times f_{i}}{f_{i,control}}$$

where *α_i_* are weight coefficients for the single studied property, which varies from 0 to 1 and their summation is equal to 1; *f_i_* is the absolute value of the given concrete mixture; and *f_i,control_* is the absolute value of the control mixture. The values of *α_i_* can be set according to their general sense and can be highly different with respect to used procedures and expected concrete exposure; a selected property can be assigned a zero value. The present discretization is a simple way to assess the durability of mixtures of various strength classes in terms of complex system of experimental procedures. This formulation follows previous research of Mostofinejad et al. [65]. 

The results of the mechanical test were not included in the DLI calculation because mechanical performance is not primarily an indicator of the durability of concrete. Six aspects were applied for the calculation of DLI, which means the value of α is approximately 0.17 for each property, if an equal value for the individual weight coefficient is applied. Equal values of α for all used properties were used by Mostafinejad et al. [65]. However, it is evident that selected properties have a different meaning, hence it was increased for selected ones and decreased for others. The motivation is the development of an easy tool for an otherwise complicated evaluation of different concrete mixtures in terms of various criterions.

The coefficient was increased in the case of freeze-thaw resistance because of structural damage and cracks propagation which cause the corrosion of reinforcement. The same reason was applied in the case of the accelerated carbonation test. A slightly increased value was applied for the chloride migration test, which makes sense especially in the case of transport infrastructure in temperate and subarctic zones. The gradual ingress of chloride ions could cause the corrosion of the reinforcement and simultaneously could contribute to alkali-silica reaction (ASR). On the other hand, the coefficient was reduced in the case of ISAT because the permeability of the concrete was partially taken into the consideration in the case of chloride migration. In addition, surface damage such as surface scaling is easily detected and could be operatively repaired. Equally, the results of the water penetration test are determined by the concrete permeability and this test could have a lower resolution in the case of mixtures with porous aggregate. The sense of the shrinkage was reduced as well, because the presence of cracks is partially taken into consideration during the design of structures by the increased amount of steel rebars. The proposed values of coefficient α are presented in Table 5.

## 3. Results

The performed experimental program was focused on the evaluation of the new concept of the concrete mix design which incorporates active mineral additives. The control mixture was based on the binary binding system consisting of Portland cement and limestone filler. The original binder was gradually replaced by fly ash. However, the replacement was conducted in terms of the amorphous phase content of FA participating in the pozzolanic reaction. The inert (crystalline) part of FA was compensated by the decreased dose of the limestone filler. The assessment was based on the durability relating procedures and prediction of service life.

### 3.1. Fresh Concrete

It is necessary to carry out a set of procedures on the fresh mixture to declare its self-compacting ability. Hence, three main test procedures evaluating the rheology properties of the SCC mixture were conducted, and the results are summarized in Table 6. Since determination of frost resistance of hardened concrete was scheduled, and the content of air in the fresh mixture was measured as well. The efficient content of air in fresh conventional concrete is at least 4.0% [31] to prevent deterioration due to internal stresses caused by ice formation. Air-entraining serves in hardened concrete for the nucleation of ice during exposure to freezing-thawing [66]. Generally, incorporation of various supplementary cementing materials increases the content of air in the fresh mixture, if they are applied as cement replacement [67,68,69]. The realizing of air bubbles inside the concrete caused by smooth FA particles is reported in [70]; the higher is the FA additive, the higher is the air content in fresh mixture. On the other hand, the stability of air bubbles up to concrete setting is a crucial aspect, which can be improved by prolonged mixing [68], mix proportions [67,71], chemical admixtures [72,73] and other special procedures [74]. Generally, higher content of air is caused by higher amounts of fines [75]. Nevertheless, a slight increase is evident in the air content for mixture with 10% and 20% of FA. The highest applied replacement exhibited a negligible decrease in air content in comparison with the control mixture. It was probably caused by the reduced stability of obtained air bubbles, which corresponds with the conclusions of Puthipad et al. [67], who declared this tendency with increasing amount of fly ash. In addition, it is necessary to note that, the higher was the dosage of fly ash, the lower was the dosage of limestone filler, hence the gradual increase of air content in studied mixtures is not necessary. The results of slump-flow, J-ring and L-box tests, which exhibited similar trend, show the reduction of measured properties of fresh mixture in case of FA-30. On the other hand, obtained values of air content in the fresh mixture could be assessed as similar, which is logical because of the constant content of mineral additives. Fly ash is favorable to apply because air-entraining contributes positively to the viscosity of the fresh mixture [70,76], which is reflected by increased workability. These findings correspond well with the obtained results of L-box test. Additionally, particles of fly ash create a ball bearing effect, thus the resulted concrete achieves better particle packing and workability [67,77]; however, higher finesses of the fly ash marginally increase the flow of the fresh mixture [78]. The results describing workability of fresh mixtures exhibited non-linear trend, which was probably caused by changing proportion of used binder component. In terms of workability, mixtures FA-10 and FA-20 improved this property, indicating suitable proportion of binder components. However, FA-10 having PC:FA:LS proportions 71.0%:13.1%:15.9% seems to be optimal.

### 3.2. Hardened Concrete

#### 3.2.1. Mechanical Properties

**Figure 3:**
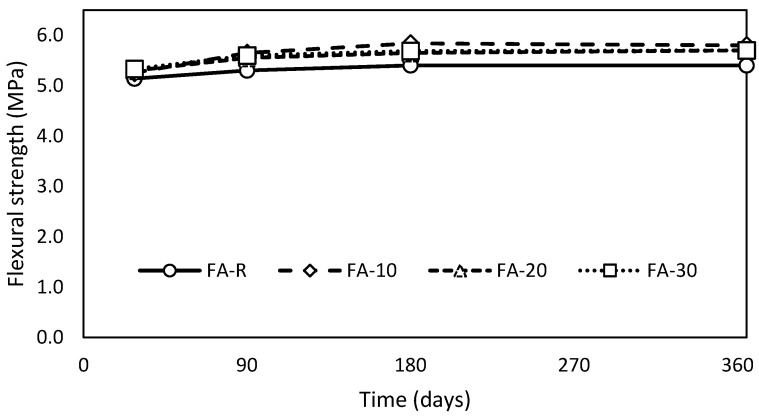
Evolution of flexural strength in time.

Mechanical properties of studied mixtures were investigated in terms of flexural strength and compressive strength after 28, 90, 180 and 360 days. All studied mixtures exhibited a gradual increase of flexural strength in time (Figure 3). Atypically, control mixture reached the lowest total values of flexural strength during the entire experiment. Esquinas et al. [79] carried out a similar research program; however, the control mixture with siliceous filler exhibited higher flexural strength and gradual incorporation of fly ash led to proportional strength decay. Similar conclusions were obtained by other researchers [24,80]. Better compaction of mixtures with fly ash, documented by better workability, is probably the reason for this behavior, especially in the case of FA-10 and FA-20. Mixture FA-30, despite exhibiting similar workability to the control mixture, except L-box test, had lower content of air in fresh mixture. In addition, mixtures without SCM often exhibit brittle mode of the rupture, which can lead in combination with lower compaction to systematic decay of flexural strength, which is very sensitive to concrete homogeneity. There is no simple explanation of this unexpectable behavior; it is probably the combined effect of other factors such as shrinkage. Mixture FA-30 reached up to 180 days better values of flexural strength than FA-20, but the negligible differences are on the boundary of measuring uncertainties.

**Figure 4:**
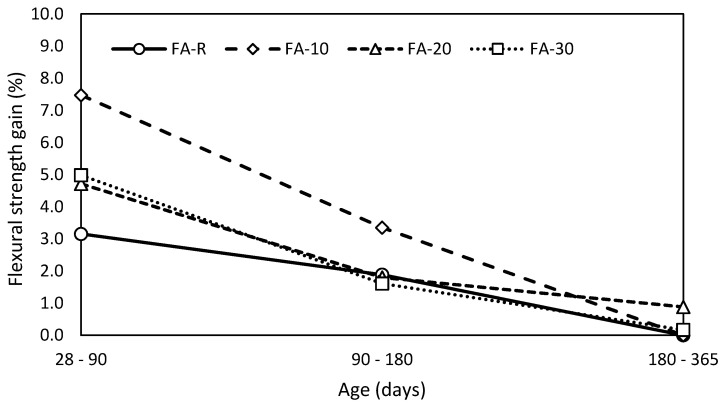
Gain of flexural strength in time.

The ongoing pozzolanic reaction is clearly shown in Figure 4, where the gains of flexural strength are expressed in time. The reaction kinetics is lowest for the control mixture and the highest for the FA-10 mixture with 10% fly ash; additional increase of fly ash content reduced the initial evolution of flexural strength. It seems that FA-20 and FA-30 are on the verge of convenience for such formulated mixtures in terms of flexural strength. Previous research works [24,25] indicated that approximately 35 wt.% of fly ash performs limiting level, which guarantees comparable properties with use of Portland cement. However, conducted binder design decreases the applicable level of fly ash utilization below 20 wt.%. The lowest gains of the control mixture are caused by the used cement CEM I 52.2 R, which exhibits rapid evolution of mechanical performance in early stages. It can achieve nearly 100%, and more than 90% of 90-day values already after 28 days of curing. The increase of flexural strength of all studied mixtures was negligible after 180 days of curing. These results correspond with similar works [81,82].

**Figure 5:**
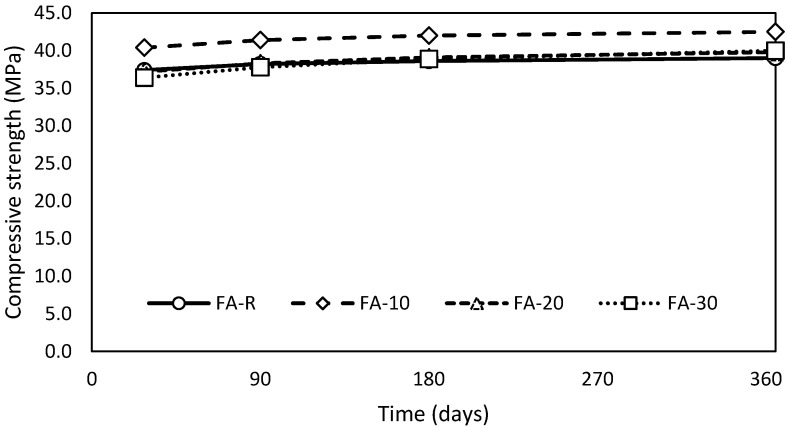
Evolution of compressive strength in time.

Compressive strength evolution in time followed a growing trend from the long-term point of view (Figure 5) in comparison with the flexural strength. The FA-10 mixture exhibited the highest total values of compressive strength in time; the curve of the progress is significantly shifted in comparison with other mixtures, by approximately 3 MPa. The remaining mixtures achieved similar results in time; however, after 180 days, the mixtures incorporating fly ash obtained higher compressive strength than the control one due to the pozzolanic reaction of the fly ash, which corresponds with the results in [77]. Individual mixtures exhibiting better workability reached improved results of compressive strength; thus, FA-10 presents the optimal ratio of used binder components. However, this optimal ratio is valid only for mixtures with given proportions of aggregate. Duran-Herrera et al. [45] conducted thorough research of the ternary binding systems consisting of PC, limestone powder and fly ash of various proportions. They reported that the presence of limestone powder positively contributed to the nucleation of hydrates during the pozzolanic reaction. The increase of internal space due to limestone powder application and subsequence precipitation of hydrates was confirmed by other authors who studied systems of PC-FA and nano-CaCO_3_ [29,83], PC-FA and ultrafine FA [84] or PC-FA and metakaolin [72]. On the other hand, De Weerdt et al. [82], in terms of thermodynamic modeling and experimental verification, described that calcium carbonate affected the transformation of AFt to AFm. This effect is observable especially in the presence of fly ash due to dotation of the alumina phases. However, valid regulations applied in praxis consider limestone as an inert mineral additive. The suitability of the FA-10 composition is also well documented by the shift of compressive strength over time. 

**Figure 6:**
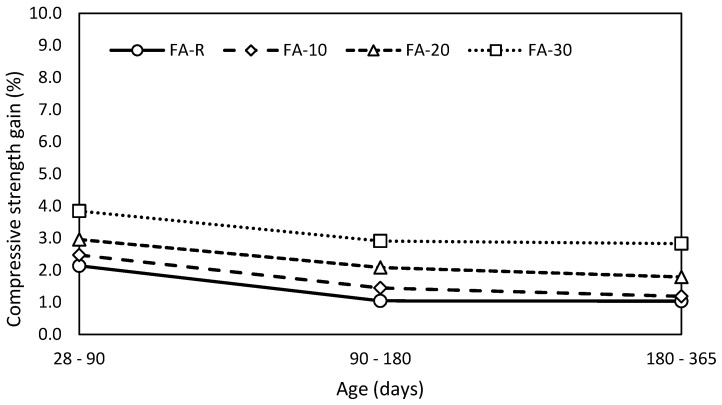
Gain of compressive strength in time.

The gradual gains of compressive strength (Figure 6) show that the increase of compressive strength in time is proportional to the fly ash incorporation. These results correspond well with the conclusions of De Matos et al. [77], who reported a negligible strength gain of the control mixture and mixtures based on the ternary binder (PC-FA-MK) after 180 days of curing. However, they reported a significantly higher strength gain during the initial phases of hardening (28–90 days): 44%, 26% and 20% for 60%, 50% and 40% substitution, respectively. On the other hand, they used the binary system (PC-FA), where the kinetics of the pozzolanic reaction is controlled by the concentration of Ca(OH)_2_ [12]. 

The obtained mechanical performance is very suitable from the mixture design point of view because of their very similar results. Individual mixtures exhibited very small differences in absolute values over time; however, especially the results of FA-R, FA-20 and FA-30 could be considered as equal with respect to the standard uncertainty of the mechanical properties determination. It is necessary to highlight the fact that labeling of the studied mixtures correspond with increasing content of fly ash, but concurrently with decreasing amount of finer limestone filler. Thus, the gain of compressive strength is closely related to the change of character of porous system.

#### 3.2.2. Frost Resistance

**Figure 7:**
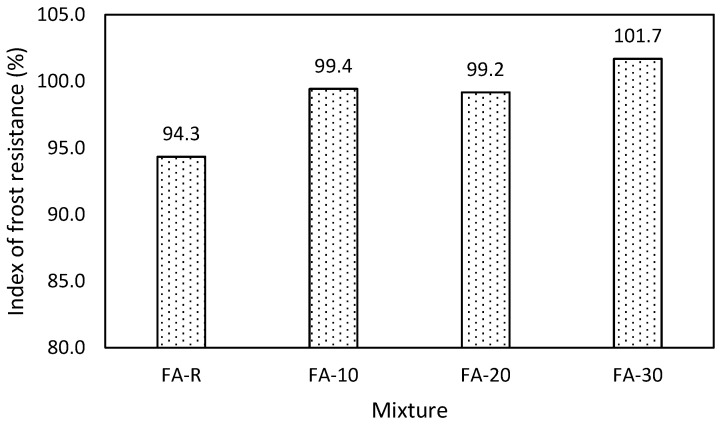
Index of frost resistance at the age of 90 days.

The index of frost resistance was determined after 90 days of curing; samples were subjected to 100 freezing-thawing cycles (Figure 7). The results demonstrate the very high resistance of all studied mixtures to freezing-thawing cycles.

No mass losses were observed during cycling. The positive effect of air-entraining due to FA presence is likely responsible for the observed behavior. That corresponds with previously published works [85,86,87]. Mixture FA-20 exhibited slightly lower value of the index of frost resistance in comparison with FA-10; nevertheless, the negligible difference recorded is probably due to variation of the measurement. The improvement of the frost resistance in the case of mixtures with fly ash was explained by the additional activation of the thus far non-hydrated particles of the mineral additive. The present assumption was confirmed in long-term research dealing with application of ceramic powder [62] by the gradual decrease of the index of frost resistance in time of mixtures which originally exhibited increase of the frost resistance. Stimulation of the binding system incorporating various mineral additives was thoroughly reported by the authors of [88]. 

#### 3.2.3. Initial Surface Adsorption Test (ISAT)

**Figure 8:**
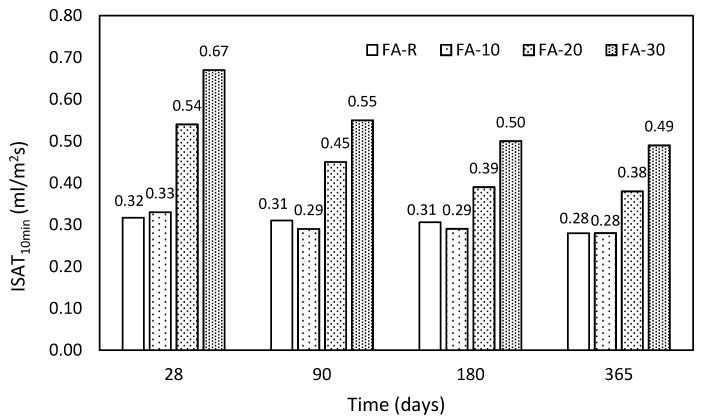
Obtained results of ISAT in time.

The results of initial surface water absorption (ISAT) are shown in Figure 8. This procedure serves as an indicator of the durability. According to the limit values introduced in Table 4, the control mixture and mixture with the lowest applied content of fly ash reached average permeability over time. At the same time, both mixtures obtained similar results, which is encouraging with respect to the applied mixture design. These mixtures did not exhibit any significant changes, but a very slight improvement over time is obvious. The remaining mixtures incorporating fly ash exhibited proportionally higher permeability, which was gradually improved to average level in time according to Table 4. This reduction of permeability was caused by the ongoing pozzolanic reaction, which corresponds with the compressive strength gains in time introduced in Figure 6. Nevertheless, the mixture with the highest content of fly ash indicates significantly increased permeability. The present aspect was reported by several authors, who dealt with SCC mixtures with high volumes of fly ash [84,89] due to increased open porosity. The essential problem of such mixtures is the fact that a large portion of fly ash was not employed by the pozzolanic reaction and from the long-term point of view will serve only as an active filler [12,89]. Hence, high cement replacement levels led to the considerable increase of permeability due to the increase of open porosity [84]. However, it is necessary to take into consideration the fact of decreasing finesses of the entire binder with increasing replacement by FA. Gradual increase of FA dosages probably led to the worse particle packing.

#### 3.2.4. Depth of Water Penetration Test

**Figure 9:**
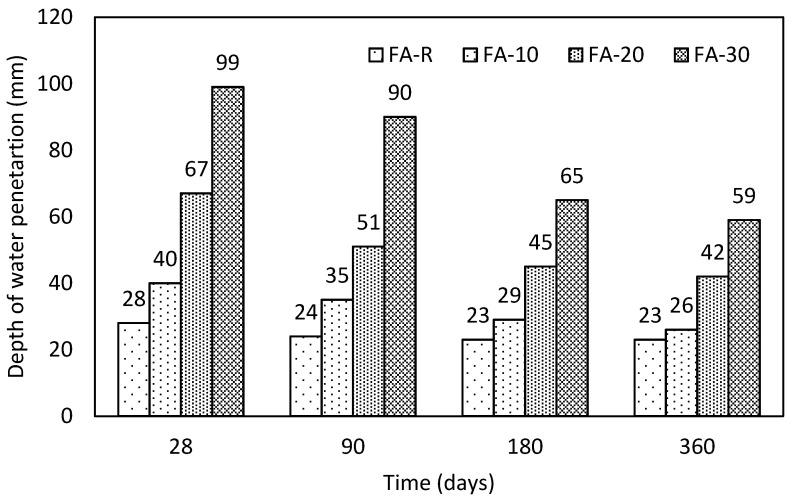
Obtained results of the water penetration test in time.

The depth of water penetration (Figure 9) increases proportionally with the content of fly ash, although a gradual decrease in time is evident for all studied mixtures. The exhibited improvement over the time for single mixtures was successively 17.9% (FA-R), 35.0% (FA-10), 37.3% (FA-20) and 40.4% (FA-30). Whereas the control mixture and the mixture with the lowest content of fly ash complied with the recommended value of water penetration according to the authors of [31] (50 mm at 28 days), the mixtures with the higher content of fly ash required prolonged curing, and the mixture with the highest applied substitution did not pass this requirement during the monitored period. That corresponds with the results of ISAT.

#### 3.2.5. Chloride Migration Test

**Figure 10:**
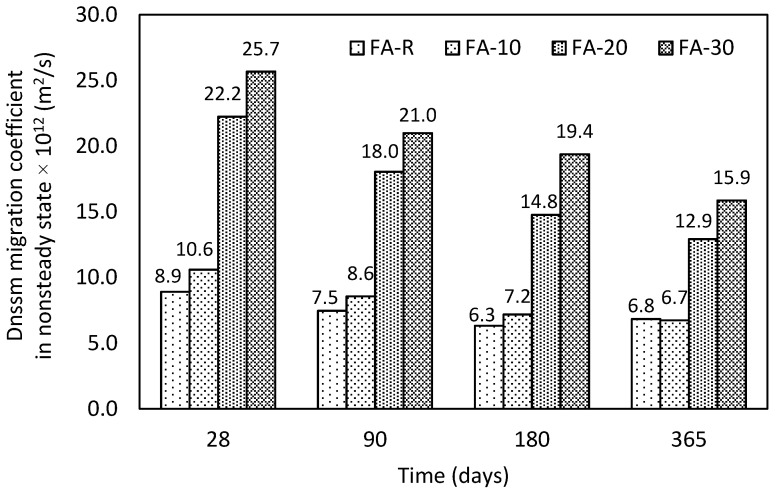
Obtained results of the chloride ion penetration test in time.

An assessment of the permeability properties in terms of chloride ion penetration was conducted in selected time intervals according to the authors of [59]. The obtained results (Figure 10) are in agreement with the results of ISAT and partially the water penetration test. All studied mixtures exhibited gradual improvement of permeability over time, especially two mixtures with the highest content of fly ash reached significant decay of permeability—FA-20 (41.9%) and FA-30 (38.1%). However, for both mixtures, the results of chloride migration test significantly shifted in comparison with the others. On the other hand, despite the higher permeability, from the long-term point of view the obtained values are still acceptable for all mixtures, namely below 16 × 10^−12^ m^2^/s. [14]. The used accelerated test and real severe conditions of a marine environment were compared by Li et al. [90], who reported good match of these two types of exposition. The obtained results of chloride migration test are partially in contradiction with the general experiences with design of marine structures, where the addition of fly ash is beneficial to reduce chloride ingress [91]. However, studied mixtures present concrete of lower strength classes with high w/b and additional dosage of limestone filler. According to Moffatt et al. [92], the w/b ratio must not exceed 0.40 to reach suitable resistance. Hence, the reduced durability of mixtures FA-20 and FA-30 could be expected. In addition, increased dosage of fly ash was compensated by the reduction of the amount of finer limestone filler; this fact has a crucial impact on the character of porous system, which was subsequently sealed over time. These results highlight the importance of the particle packing. Similar results were published by Celik et al. [93], who studied SCC mixtures based on PC-FA-LS. They reported increased chloride migration coefficient of ternary systems in comparison with the binary ones due to worsened characteristics of pore system.

#### 3.2.6. Carbonation

**Figure 11:**
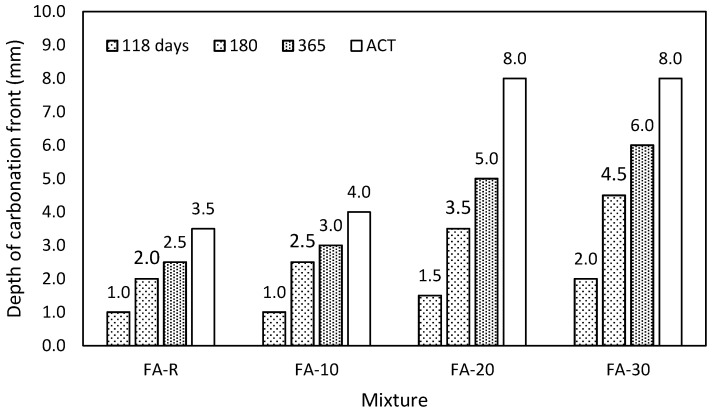
Comparison of natural carbonation in time and the accelerated test on studied mixtures.

Determination of the depth of carbonation was carried out in selected time intervals with respect to the prescribed procedure. The first test was carried out at the age of 118 days with prior four weeks drying in normal laboratory conditions. The other values determined after 180 and 365 days were achieved on the samples, which, from the age of 90 days, were kept in normal laboratory conditions. The obtained results (Figure 11) indicate that the depth of the carbonation front determined after the accelerated test is similar to the one measured after one year. The control mixture and the mixture with the lowest addition of fly ash exhibited very good resistance to carbonation. On the other hand, mixtures with increased content of fly ash showed rapid progression of the carbonation front. These results correspond with the conclusions of Singh and Singh [94], who observed the increased carbonation progression of high-volume fly ash self-compacting concrete. They also recommended the addition of metakaolin to reduce the carbonation. Similar results and conclusions were reported by Esquinas et al. [95], who studied SCC mixtures with fly ash, which exhibited increased carbonation with increasing content of fly ash; however, this effect was essentially suppressed by a small addition of silica fume. Identically effective for the improvement of carbonation resistance is the reduction of w/b and prolonged curing [42]. Da Silva and de Brito [96] reported increased penetration of CO_2_ into binary SCC mixtures incorporating higher doses of limestone filler, respectively fly ash. Thus, in this context, ternary binders seem to be more effective. The recommended dose of fly ash in the ternary system with limestone filler was up to 30% of PC. The obtained values of carbonation depth are in a good agreement with those presented in this paper since a different mix design was applied (see Table 3).

#### 3.2.7. Shrinkage

**Figure 12:**
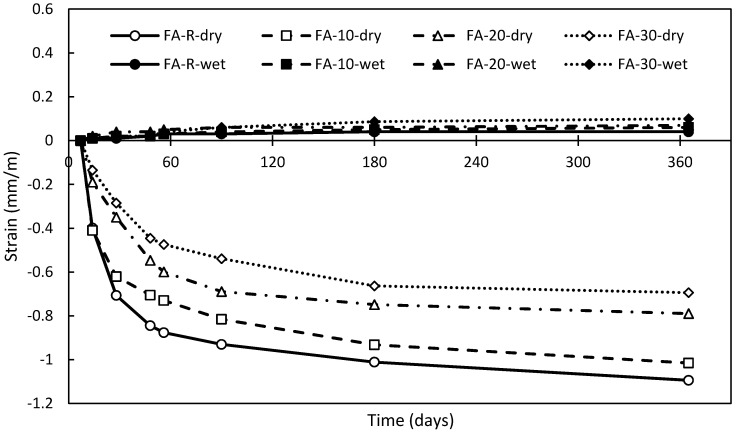
Results of shrinkage measurement of studied mixtures in time.

The obtained results of shrinkage measurement are shown in Figure 12. The reduced shrinkage with increasing amount of fly ash is well displayed. The final shrinkage after one year in laboratory conditions for single mixtures was 1.09 (FA-R), 1.02 (FA-10), 0.79 (FA-20) and 0.69 (FA-30) mm/m. That means, in comparison with the control mixture, a gradual reduction by 6.4%, 27.5% and 36.7%, respectively. The positive influence of fly ash on the resulting shrinkage is determined by the lower initial hydration kinetics of binders incorporating pozzolans, which explains the opposite trend of the binary mixture with reactive silica fume. That is in good agreement with similar works and conclusions [47,95,96]. Sets of samples cured in water exhibited slight expansion, which varied from 0.04 to 0.10 mm/m. This effect was thoroughly described by Rahimi-Aghdam et al. [97] and explained through the fixation of capillary water. 

**Figure 13:**
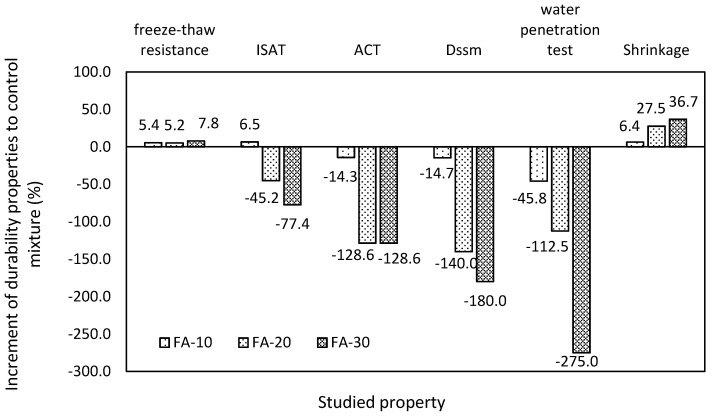
General changes in durability properties in comparison with the control mixture.

The conducted experimental program highlighted the necessity of a complex durability assessment during concrete composition design. All studied mixture exhibited gradual reduction of the quality with increasing dosage of FA in terms of all procedures relating to the permeability. It is caused by the higher finesses of used limestone filler, the dosage of which is disproportional to FA. The general evaluation of the properties in relation to the durability is shown in Figure 13, where the increment of selected procedures for mixtures incorporating the fly ash is illustrated. The presented values are related to control mixture (FA-R); values used for this presentation are properties attained after 90 days of curing. It is clearly shown that the applied mix design has a positive impact only on the shrinkage, freeze-thaw resistance and initial surface absorption for the lowest applied dose of fly ash. The remaining procedures exhibited significant decay with an increasing dose of fly ash. It is necessary to note that these techniques reflect the permeability of studied concrete, which is a property determining the final durability. 

#### 3.2.8. Durability Loss Index (DLI)

The durability loss index (DLI) was calculated for individual mixtures because of significant differences in their composition and subsequent mechanical performance. In addition, the DLI expression allows taking into consideration a higher number of factors. Two different combinations of *α* values were applied: an equal value of *α* was assigned for individual tests in the first one and *α* was modified according to the importance of individual procedure, as shown in Table 5, for the second one.

**Figure 14:**
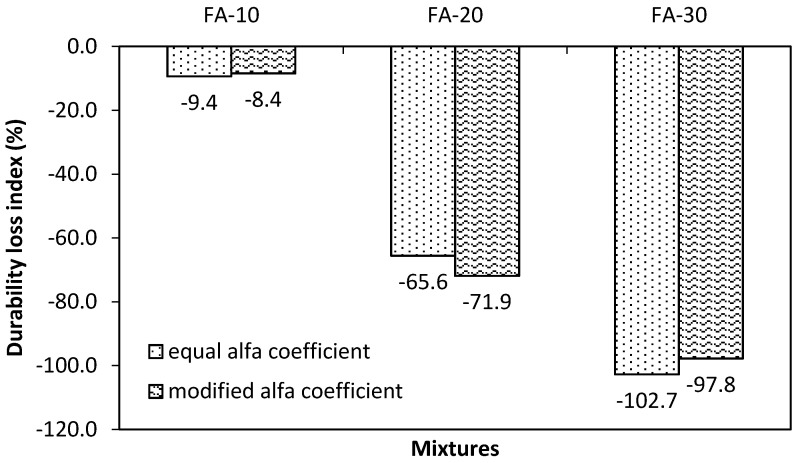
Calculated values of the durability index for single mixtures.

The calculation of DLI confirmed a similar trend for both applied combinations of the *α* value. The highest influence came from the accelerated test of carbonation, chloride migration and water penetration test, which exhibited the highest relative differences, as clearly shown in Figure 14. Changes in values of *α* for single procedures did not cause substantial differences in the final *DLI*. It can be stated that, for both studied combinations of *α*, the increasing content of fly ash in the concrete mixture proves the gradual loss of durability. However, it is necessary to note that the proposed values of *α* in this program reflected the applied set of procedures and their mutual importance with respect to expected exposure. For example, in the case of concrete structures, which are permanently immersed in water, the weight value belonging to carbonation could be set to zero.

The proposed approach for the mixture design, which takes into consideration the mineralogical properties of used fly ash, confirmed its potential only from the mechanical performance point of view. The achieved mechanical properties of FA-10 were similar to or higher than the control mixture. In relation to durability, this approach is not reliably applicable because all mixtures incorporating fly ash exhibited reduced durability performance. On the other hand, the FA-10 mixture, which from the conventional point of view contained 18.5% replacement of PC by FA, exhibited a loss of durability of up to 10% in comparison with the control mixture. These results indicate that the application of the ternary binder system (PC-FA-LS) could lead to loss of durability in the case of the SCC mixtures of lower strength classes. Based on the calculated values of DLI, it could be estimated that critical proportions are below applied mixture labeling. The lowest dosage of FA corresponds to the proportions (PC:FA:LS) 71.0%:13.1%:15.9%, and this mixture exhibited slightly reduced DLI in proposed constitution of single weight factors.

**Figure 15:**
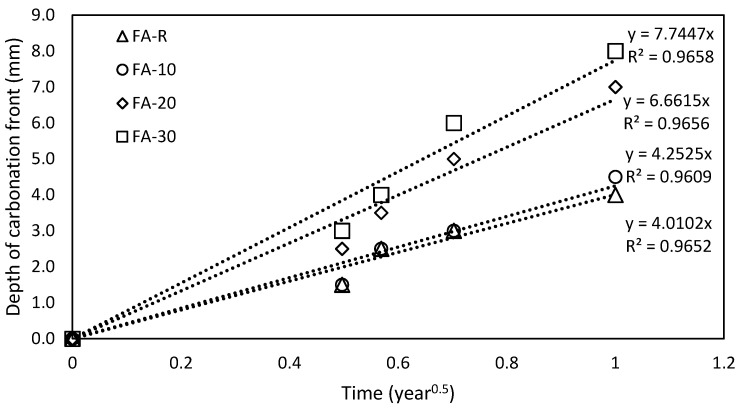
Calculated values of the durability index for single mixtures.

The received values of DLI were highly influenced by the accelerated tests results. However, these results are partially distorted by the required prolonged curing; hence, it is necessary to evaluate them from a realistic point of view, i.e., in normal laboratory conditions. For this purpose, the progress was used of the carbonation front of samples which were cured under normal laboratory conditions. The obtained values are shown in Figure 15, where the indicated values of *K_field_* (Equation (1)) are also shown. The obtained values of *K_field_* were subsequently used for the prediction of the service life of the studied concrete mixtures.

#### 3.2.9. Service Life Prediction.

**Figure 16:**
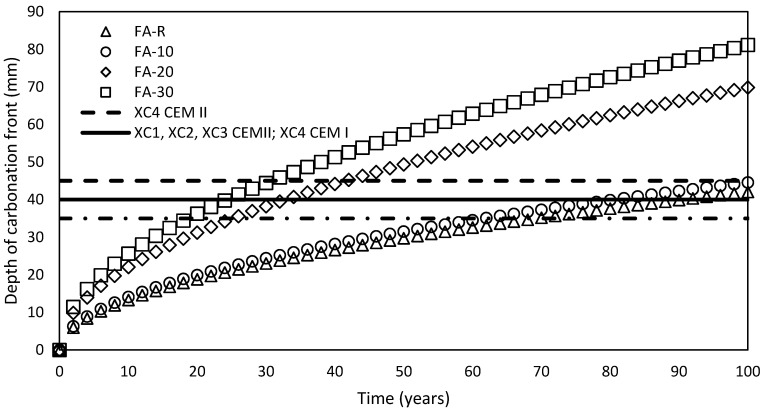
Prediction of service life according to the results of carbonation.

The prediction of service life was conducted in accordance with the model of EHE-08 [63] (Equation (2)). The requirements for increased resistance of concrete to the ingress of carbon dioxide is taken into consideration only through passive, natural, protection of concrete, which is controlled by the diffusion. Hence, the increased risks of the action of carbon dioxide and subsequent reinforcement depassivation are reflected by the thickness of the nominal concrete cover. The conducted prediction reflecting requirements of CSN EN 206-1 [31] is shown in Figure 16. The numerical output of this prediction is then summarized in Table 7.

The obtained results correspond well with the conclusions of Wang et al. [98], who studied the influence of the load on the evolution of carbonation front. Their control sets of samples manifested itself by the reduced resistance to carbonation in the case of the higher replacement level of fly ash (40%). However, the behavior of fresh mixtures has to be taken into consideration during an evaluation of the effect on carbonation of mineral additives. Singh and Singh [99] studied resistance to carbonation of SCC mixes incorporating fly ash, metakaolin and recycled aggregates. Their results indicate increased resistance of mixtures with higher doses of fly ash; nevertheless, an increased dose of fly ash improved performance of fresh mixtures, which contributed to the tightness of the hardened concrete. The physical effect of SCMs was also highlighted by Pacheco Torgal et al. [100].

The reduced resistance to carbonation of blended systems was thoroughly studied by Marques et al. [101], who compared the results of the prediction model using the results of carbonation tests with requirements of EN 206-1 [31]. Their results correspond well with the conducted experimental program that an increasing dosage of fly ash in Portland cement–fly ash–limestone filler binding system tends to reduced resistance to carbonation. 

The performed experimental program confirmed that studied SCC mixtures with higher applied replacement exhibited significantly reduced resistance to the ingress of chlorides, carbonation and water penetration, crucial aspects of steel corrosion. It was caused by the worse particle packing, because the used FA was coarser in comparison with LS. Unfortunately, SCC mixes of lower strength classes are currently favored by contractors; despite suitable mechanical performance and freeze-thaw resistance, these modern mixes introduce potential risks. The results of conducted work indicate that SCC mixes of lower strength classes are exhibiting higher permeability. The replacement exceeding 30 wt.% presents risks in relation to significant decay of pH of hardened concrete causing potential corrosion of embedded steel [27,102].

## 4. Conclusions

The present experimental program was focused on the study of SCC mixes incorporating fly ash and limestone filler. The new method of binding system design, which takes into consideration the amount of amorphous phases in studied mineral additive, was studied. The inert part of fly ash was compensated by the limestone filler, i.e., the studied SCC mixtures were based on the ternary binder system. The assessment of the new concept of binder formulation was carried out in terms of properties of fresh and hardened concrete. However, the main aim of the research was the mix design verification in terms of the long-term durability. For this purpose, a complex system of tests was conducted and the evaluation was carried out by using durability loss index, which allows general comparison by using results of various procedures. The obtained conclusions are summarized as follows:

(1) Mixtures designed in terms of proposed concept reached similar workability as control mixture; all mixtures satisfied self-compacting criteria without additional dosage of chemical admixtures. However, the lowest applied fly ash replacement exhibited a slight increase in workability due to higher air-entraining.
(2) The proposed design of the binding system ensures similar or slightly higher values of compressive strength, especially from the long-term point of view.
(3) An increase of mechanical performance was negligible after 180 days of curing. Mixtures containing fly ash exhibited higher flexural strength in comparison with the control mixture. The lowest dose of fly ash led to the increment of compressive strength by approximately 10%, while additional increase of fly ash replacement maintained compressive strength equal to the control mixture.
(4) The gradual increase of cement replacement by fly ash led to a significant reduction of shrinkage and improvement of freeze-thaw resistance.
(5) Replacement by fly ash in proposed procedure indicated a significant increase of permeability controlling carbonation, water penetration and chloride migration, which increases the risk of embedded steel corrosion. It was caused by gradual substitution of finer limestone filler by coarser fly ash.
(6) SCC mixture with the lowest applied cement replacement exhibited reduced, but still acceptable, durability performance. Despite increased water penetration, this mixture obtained similar absolute values in terms of chloride migration and carbonation depth.
(7) Final derived values of DLI of individual concrete mixtures were dominantly affected by the results of carbonation tests.
(8) DLI offers a useful tool for the evaluation of concrete mixtures in terms of various criteria. In addition, the choice of the values of weight factors for single criterion allows the assessment of different exposures or experimental setups. The calculation of DLI indicates that optimal proportion of ternary binding system should kept the level of fly ash for these type mixtures below 13 wt.%.

The present experimental research highlighted the necessity of the inclusion of durability aspects into the concrete mix design. Modern SCC mixtures studied in the program exhibited significant loss of durability through increased permeability. Active mineral additives offer great potential for sustainable structural engineering; however, it is necessary to focus further research on the long-term durability provision

## Figures and Tables

**Table 1:** Chemical composition and properties of the used binder component.

Binding Component	SiO_2_ (%)	Al_2_O_3_ (%)	Fe_2_O_3_ (%)	CaO (%)	MgO (%)	Na_2_O (%)	SO_3_ (%)	K_2_O (%)	TiO_2_ (%)	LOI (%)
Cement	18.1	6.4	2.4	64.9	1.0	0.3	4.9	1.2	0.8	1.1
Fly ash	52.4	35.9	4.9	1.2	0.8	-	0.2	1.4	2.4	2.8

**Table 2:** Mineralogical composition of used fly ash.

Amorphous Components	60.0
Quartz	7.9
Mullite	28.8
Magnesioferrite	1.5
Anhydrite	0.7
Hematite	1.2

**Table 3:** Composition of studied SCC mixtures (kg/m^3^).

Components	FA-R	FA-10	FA-20	FA-30
CEM I 52.5 R (kg/m^3^)	355.0	319.5	284.0	248.5
Milled limestone (kg/m^3^)	95	71.3	47.7	24.0
Fly ash (kg/m^3^)	0	59.2	118.3	177.5
Sand 0–4 mm (kg/m^3^)	1030	1030	1030	1030
Crushed agg. 4–8 mm (kg/m^3^)	270	270	270	270
Crushed agg. 4–8 mm (kg/m^3^)	400	400	400	400
Plasticizer (kg/m^3^)	3.8	3.8	3.8	3.8
Retarding agent (kg/m^3^)	1.5	1.5	1.5	1.5
Water (kg/m^3^)	190	190	190	190
PC-FA-LS (%)	79.0-0-21.0	71.0-13.1-15.9	63.1-26.2-10.6	55.2-39.4-5.3
w/(PC + FA + LS)	0.42	0.42	0.42	0.42
w/(PC + FA)	0.54	0.50	0.47	0.45

**Table 4:** Indicative assessment of concrete permeability using ISAT [56,57].

**ISAT_10 min_ (g·m^−2^s^−1^)**	**Permeability Classification of Concrete**		
	**Low**	**Average**	**High**
	<0.25	0.25–0.50	>0.50

**Table 5:** Proposed values of weight coefficient α (-).

Freeze-Thaw Resistance	ISAT	ACT	Dssm	Water Penetration Test	Shrinkage
0.25	0.10	0.25	0.20	0.10	0.10

**Table 6:** Properties of the fresh mixture.

Properties of Fresh Concrete	FA-R	FA-10	FA-20	FA-30
Air content (%)	5.0	5.3	5.2	4.9
Slump-flow test (mm)	620	690	650	620
J-Ring test (mm)	590	680	620	590
L-Box test (-)	0.92	0.95	0.96	0.95

**Table 7:** Estimated depassivation time and minimum cover required.

Mixture	Depassivation Time (Years)			Minimum Cover for 100 Years (mm)
	CEM I XC1, XC2, XC3	CEM I XC4 CEM II XC1, XC2, XC3	CEM II XC4	
FA-R	69	91	115	42
FA-10	62	81	102	45
FA-20	25	33	42	70
FA-30	19	24	31	81
